# Social Network Analysis of the Schistosomiasis control program in two local government areas in Oyo state, Nigeria: Insights for NTD elimination plans

**DOI:** 10.1371/journal.pntd.0011266

**Published:** 2023-04-07

**Authors:** Adeola Onasanya, Jo van Engelen, Opeyemi Oladunni, Oladimeji Oladepo, Jan Carel Diehl

**Affiliations:** 1 Department of Sustainable Design Engineering, Faculty of Industrial Design Engineering, Delft University of Technology, Delft, The Netherlands; 2 Department of Public Health, Adeleke University, Ede, Nigeria; 3 Department of Health Promotion and Education, Faculty of Public Health, College of Medicine, University of Ibadan, Ibadan, Nigeria; Texas A&M University College Station, UNITED STATES

## Abstract

**Background:**

Schistosomiasis is one of the neglected tropical diseases targeted for elimination by 2030. Achieving disease elimination requires collaboration between stakeholders, country ownership and the involvement of community-level stakeholders. The state of stakeholder relationship determines the ease and timeliness of meeting disease elimination targets. Mapping stakeholder relationships is critical for assessing gaps in the schistosomiasis control program implementation, and providing a roadmap for improved stakeholder cohesion. The study aimed to measure the cohesiveness of the contact, collaboration and resource-sharing networks, across 2 local government areas in Oyo state, Nigeria.

**Materials and methods:**

This study used a Network Representative design for Social Network Analysis (SNA). The study was conducted within Oyo state, Nigeria using 2 Local Government Areas (LGAs): Ibadan North (urban) and Akinyele (rural). Stakeholders were identified using a link-tracing approach. Data was collected using Qualtrics software from stakeholders across the state, local government, healthcare, academia, and non-governmental organizations. Data was analysed using Gephi software for network cohesion across the three networks.

**Results:**

The social network analysis revealed high clustering and low density across the three networks implying low cohesion across multiple stakeholder categories. The contact and collaborative networks were the most active with the lowest level of cohesion seen in the resource-sharing network. Stakeholders were more active in the rural LGA than the urban, and stakeholders within the organized governance and public health system were the dominant actors in the schistosomiasis control program.

**Conclusion:**

The low cohesion, high clustering and low network density among stakeholders within the schistosomiasis control program should be addressed in other to drive innovation and meet the WHO schistosomiasis elimination target.

## Author summary

Schistosomiasis is one of the neglected tropical diseases which is planned for elimination by 2030. Eliminating the disease requires collaboration among important government, community, healthcare providers and non-governmental organizations representatives working on schistosomiasis. The current level of cooperation and collaboration among these representatives in Oyo state, Nigeria is unknown. This study aimed to measure the extent and strength of collaboration among these representatives looking at three relationships: contact, collaboration and resource sharing activities using social network analysis method. Undertaking this research will support a better understanding of barriers against meeting the disease elimination deadline. The social network analysis found that the three relationships patterns had considerable clustering among representatives and few connections, signifying low interconnectedness across several representative groups. The contact and collaborative relationships were the most active, with the resource-sharing network having the lowest amount of interconnectedness. Representatives were more active in rural areas than urban, and representatives in the organized government and the public health system were the most active actors in the schistosomiasis control program. The schistosomiasis control program’s representative’s poor connectedness, unbalanced relationships patterns should be addressed in order to stimulate improvement and reach the WHO schistosomiasis elimination objective.

## Introduction

Schistosomiasis is a Neglected Tropical Disease (NTD) endemic in 78 countries, with more than 90% of people infected with the disease living in Africa [1]. Within several national health systems, there is a focus on disease control and elimination through schistosomiasis control programs [2]. The focus of the schistosomiasis control program is country-specific, however, strategies for control are focused on a mix of policies including Water, Sanitation and Health education (WASH) activities, preventative chemotherapy in form of Mass Drug Administration (MDA), environmental control and disease surveillance [3,4]. These measures alone appear insufficient for schistosomiasis elimination since many countries are yet to eliminate the disease.

The WHO has set new targets for NTD control and elimination for 2021–2030 with schistosomiasis being planned for elimination by 2030. The pillars for meeting these targets include accelerating programmatic action, intensifying cross-cutting approaches, and changing operating models and culture by increasing country ownership [1]. These pillars can only be operationalized by stakeholders at the global, regional, national and subnational levels; and sustainable progress will largely depend on these stakeholders’ abilities to collaborate to achieve these aims. Collaboration between stakeholders can take different forms and include elements of contact or communication, resource support and collaborative activities [5–7] all leading to stakeholder cohesion. Collaboration is usually preceded by stakeholder identification and engagement.

To achieve the WHO endgame, there is a need to engage all stakeholders within the schistosomiasis control stakeholder network for several reasons. First, stakeholder engagement is required for an effective definition of gaps and challenges within varying contexts. Second, proffering and operationalizing solutions to the gaps can only be effective if stakeholder buy-in is guaranteed. Finally, the development and diffusion of innovative practices and products such as new diagnostics to drive change and ensure disease elimination is possible if stakeholders collaborate. It is known that stakeholder engagement is shaped by various factors which include cultural, social and political context, and resource limitations [6]. Stakeholder engagement requires equitable contribution from all relevant stakeholders supported by a mutual understanding of roles and support as stakeholders have varying levels of time, resources, and skills [5] available to achieve the control and elimination of schistosomiasis.

The WHO has also emphasized the need for the integration and streamlining of various aspects of the control program into the healthcare system and the need for sectoral collaboration. However, it is unclear how the collaboration will be led as the roles of stakeholders have to be clearly defined to achieve the NTD elimination targets. Therefore, it is important to identify current gaps in the stakeholder collaborative network within the schistosomiasis control program. This is a prerequisite to proffering solutions which will lead to enhanced coordination, communication and collaboration for meeting disease elimination targets.

Research on stakeholders within the schistosomiasis control program in Nigeria has identified different stakeholder categories [8]. However, it is unclear how these stakeholders interact and if there are differences and similarities between interactions in both rural and urban contexts. It is known that strong coordination among stakeholders promotes role clarity and fosters inclusiveness, which strengthens collaboration leading to the timely meeting of targets [7]. Pillars 1 and 3 of the WHO endgame focus on collaboration and alignment among stakeholders not only at national or global levels, but more importantly at sub-national levels and the local government/municipal levels [1]. Furthermore, the involvement of community structures particularly community leaders and civic leaders including patient groups and people living with NTDs, all have a role to play in community buy-in and cooperation with the local NTD structures [9] leading to more disease awareness and sustained behavioural change.

As such, studying stakeholder relationships can give insights into the dynamics of collaboration among stakeholder groups to reveal gaps and opportunities for stronger collaborative actions to meet the WHO target. One of the ways stakeholder relationships can be studied is through Social Network Analysis (SNA). SNA, a type of systems research, is a method of investigating stakeholder influence, connectedness and cohesion within a network [10]. The analysis of social network structures is an offshoot of graph theory and promotes a way to understand stakeholders’ influence based on their position within a network. Studying network dynamics can help build an understanding of specific relationship dynamics that can affect the operations of stakeholders, alongside the strength and importance of different stakeholders within these networks. These insights can provide opportunities to build trust, improve communication and information flow, increase collaboration, and maximize the potential of stakeholders thereby improving the whole system [10,11].

Social networks are known to be both multi-layered and multi-relational based on the social characteristics of the individual stakeholders, the relationship characteristics between stakeholders and the system or organization wherein they function. [12]. This means that the relationship between the same stakeholders can vary based on the relational characteristic being explored leading to multiplicity of relational data.

One way to address the issues of the multiplicity of stakeholder relationships without using a reductionist, combinatorial approach to multiplex data on relationships [13,14] is to use the Network Representative method (NetRep method) [10]. The NetRep method is a newly developed methodology that enables efficient data collection by performing intensive sessions with representative actors/stakeholders and using non-parametric analysis in form of graphical interpretation to explore the patterns among multiple kinds of stakeholder relationships [10]. This simplifies explaining stakeholders’ relationships and avoids extensive mathematical modelling interpretation. The NetRep method ensures the compactness of the dataset without sacrificing the quality and depth of the results [10]. This method was developed and used to visualize how the multiple relationships impact each other and explore the specific characteristics of different networks essential to the analysis of a regional governance system [10]. This system is analogous to the healthcare system in Nigeria in terms of role multiplicity and interactions between people, processes, products/services, and organizations all enmeshed within the sociocultural paradigm of strong informal relationships within the society. We will be exploring collaborative processes including contact patterns, resource support and linkages among the stakeholders within the schistosomiasis control program in Oyo state.

There are several studies including systematic reviews on social network analysis among healthcare organizations in developed countries [15–21], and a few studies on organizational SNA from a developing country setting [22–25]. Within the African context, there are no known studies exploring the relationships between the stakeholders within the local NTD network generally, the stakeholder network of the schistosomiasis control program specifically, and how the state of these relationships can affect disease control and elimination. As such, this study will contribute to the literature on the use of SNA in the healthcare context within the sub-Saharan African context, as well as an understanding of relational factors that impact schistosomiasis disease control policies.

## Methods

### Ethics statement

Ethical approval was given by the University College Hospital, Ibadan/University of Ibadan UCH/UI Joint Ethical Review Committee (UI/EC/21/0100). Written Informed consent was given by all interviewed participants.

### Study design

We used a comparative quantitative research design to assess the stakeholder relationship patterns within the schistosomiasis control program in 2 Local Government Areas (LGAs) in Oyo state, South-West Nigeria.

### Study setting

Oyo state was selected based on previous research [8] carried out outlining the stakeholders’ roles within the schistosomiasis control landscape, and the state’s moderate prevalence of schistosomiasis. The study data was collected at 2 levels: state and local government levels. 2 Local Government Areas (LGAs): Ibadan North (urban) and Akinyele (rural) local government areas were purposively selected based on previous work [8] and previously established relationships with stakeholders in these LGAs. Choosing an urban and rural LGA was to aid comparison and document differences between stakeholder behaviour in different contexts within the same state.

### Participant selection

Participant selection was based on a link-tracing approach. Four categories of stakeholders were sampled based on work done by [8]. These include stakeholders within both the formal health system (public and private) and the 3 levels of healthcare (primary, secondary and tertiary), stakeholders within the organized health governance who are in charge of local programs, stakeholders within the policy and financing space which includes Non-Governmental Organizations (NGOs) and developmental agencies that support the schistosomiasis control program within the state and stakeholders in academia who are working in the neglected tropical disease field. The roster of stakeholders generated from work done by Onasanya et al. (2020) was used to compile the network roster. A snowball approach was incorporated during data collection to validate as well as identify other stakeholders who may be important in the schistosomiasis control program. In total, 33 stakeholders were identified and 32 stakeholders were interviewed (Table 1). One stakeholder did not respond to interview requests.

**Table 1:** Stakeholder distribution.

Stakeholder category	Stakeholder level			
	State level	Local Government	Public health facility	Private health facility
Healthcare	Head of Laboratory services [1]	*Medical Officer of Health (MOH) [2]	*Primary care*: CHEW [2], CHO [1], Nurse [4], Doctor [3], Laboratory Technician [2] *Secondary care*: Laboratory scientist [1] *Tertiary care*: Doctor [1]	*Private laboratory*: Laboratory Scientists [3] *Private hospitals*: Doctors [1]
Governance	State Disease Surveillance and Notification Officer (DSNO) [1], Neglected Tropical Disease officer (NTD) [1], Researcher [1], Primary Health Care Director [1]	LG Disease Surveillance and Notification Officers [2], LG NTD officer [1]+	++	N/A
**Policy/financing	WHO [1], CDC [1], Federal Monitoring and Evaluation Officer [1]	-	N/A	N/A
Academia	Researcher [1]	-	N/A	N/A

*MOH carries out policy, healthcare and governance functions + one DSNO has a dual role (DSNO+NTD officer)

**policy/ financing stakeholders work within the state and not for the state

++ Doctors and CHO were both heads of facilities and healthcare workers

### Data collection

The number of respondents was largely similar across the 2 LGAs. 10 stakeholders were interviewed for Akinyele LGA and 9 stakeholders were interviewed in Ibadan North LGA. These stakeholders include primary healthcare workers in both public and private facilities and those within the LGA NTD governance structure. Other stakeholders were those within the state NTD governance structure, healthcare workers at the secondary and tertiary level of care and stakeholders within policy, financing and academia.

### Procedure

Identified stakeholders were approached after the informed consent. The Qualtrics software listed all previously identified stakeholders for the schistosomiasis control program. Participants were asked to list additional stakeholders and validate the stakeholder roaster. Thereafter, participants were asked to select the top 10 stakeholders that were important for the schistosomiasis control program. Questions relating to contact, linkage/collaboration, and resource support were elicited about each participant’s top list of stakeholders within a one year period (Table 2).

**Table 2:** Network relationship terms.

Network	Meaning	Constituent/types
Contact	Refers to any form of contact/communication activity between stakeholders.	Response: YES/NO Frequency: Never Annual Biannual Quarterly Monthly Weekly Daily Type: Phone calls, Meetings, Emails, Others
Linkage/collaboration	Refers to the degree of collaboration between stakeholders	Response: Not linked, Communication, Cooperation, Coordination, Collaboration, Partnership, Fully linked
Resource support	Refers to a stakeholder providing resource support to another stakeholder	Response: Yes/NO Type: Financial, Technical, Political, Training, Professional, IT, Ideas, Material

### Tools

The quantitative survey (Table 2) was designed by the investigators after a literature review and going through previously collected data [8]. A face and content validity review was conducted in consultation with public health experts to ensure that respondents fully comprehend the research questions and questions that address research objectives. Data was collected using the Qualtrics survey software 2021.

### Data analysis

Data was coded and cleaned using Microsoft Excel software. Stakeholders were coded numerically as nodes while relationships were coded as edges. Analysis of the relationships between stakeholders was mapped using Gephi software version 0.9.6 202206221744. Data was analyzed by the local government area for contact, linkage, and resource support patterns at the network and stakeholder/actor levels (Table 3). Network levels indices calculated include network diameter, density and clustering. Actor-level indices calculated include degree and betweenness centrality. Data was visualized using the Fruchterman-Reingold layout which emphasizes complementarities.

**Table 3:** Definition of social network terms.

Network level indices	
Network diameter	Network diameter is the average space or separation between actors. It is the shortest distance between the two most distant stakeholders. Networks with low diameter are cohesive networks with little clustering. It measures the efficiency of information flow within the network
Average degree	This is a measure of the overall connectivity of the network
Density	This is the number of current connections within the network divided by the maximum number of connections possible. Measurement ranges from between 0 and 1. Destiny can be computed as low (<0.3), moderate (0.3–0.5), or High (>0.5) [18]
Average clustering coefficient/transitivity	This measures the degree of intra-group cohesion within a network. Networks with high clustering indicate that stakeholders are connected in dense pockets of interconnectivity. Clustering can accelerate intra-group behaviour change.
**Actor level indices**	
Degree centrality	This is the number of links incident to a certain node/actor. It is a measure of the involvement of the stakeholder in the network It can be used to find highly connected stakeholders who are more likely to have access to information and influence others’ decisions
Betweenness centrality	This measures the actors/ stakeholders who act as ‘bridges’ between other stakeholders in the network. It is used for finding the individuals who are gatekeepers and can influence the flow of information and resources around a system. Since they connect different groups of stakeholders, they usually have multidisciplinary knowledge

After data analysis, 2 central stakeholders within the network were shown the network graphs to comment on network structure, relationship patterns and stakeholder list completeness as a means of data validation. The stakeholders agreed on the completeness of the network, relationship patterns and stakeholder list.

## Result

### NTD Network Cohesion

**Fig 1:**
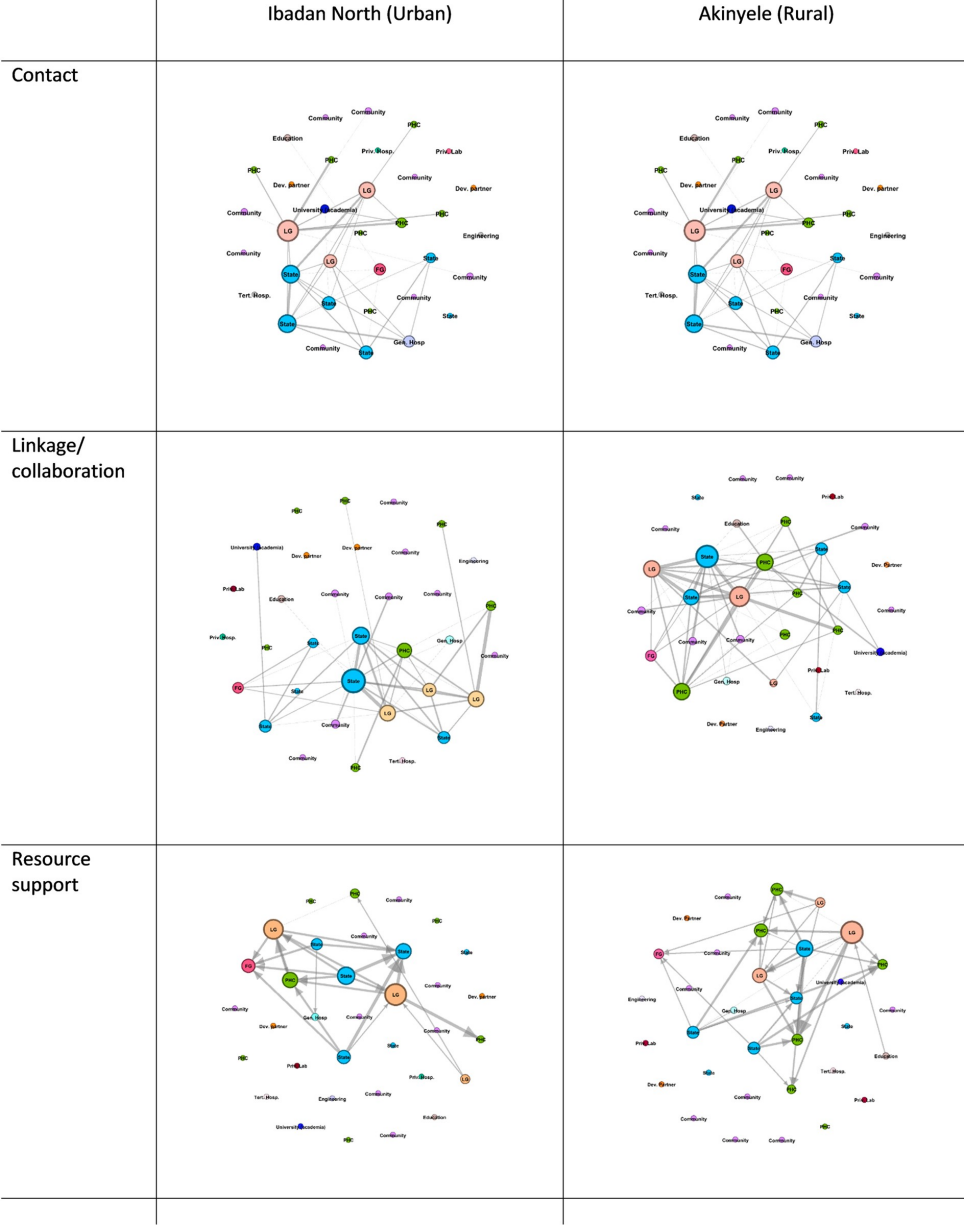
Degree centrality across three networks. Key: LG: local government, FG: Federal Government, State: state government, PHC: Primary Health care centre, Priv. hosp: private hospital, Priv lab: private lab, Dev Partner: Developmental partner, Tert. Hosp: Tertiary hospital, Gen Hosp: General hospital (secondary care).

Table 4 shows the number of ties between stakeholders, network diameter, density and average clustering coefficient of the contact, collaboration and resource support networks of the 2 LGAs. Akinyele LGA has a higher number of ties across the three networks. The network diameter for the 2 LGAs across the network relationships was similar. The density of the three network relationships was low in both LGAs. However, the highest density was seen in the collaboration network in Akinyele (0.117) while the lowest density was seen in the resource support network in Ibadan North (0.021). The clustering coefficient was highest within the collaboration network in Akinyele LGA (0.648) and lowest within the resource support network (0.05) in Ibadan North. All multiplex relationships show the formation of strong connections between the local government, state, federal government and primary healthcare centres (Fig 1).

**Table 4:** NTD network cohesion.

	Contact		Linkage/collaboration		Resource support	
	IBN	Akinyele	IBN	Akinyele	IBN	Akinyele
Number of relational ties	41	58	50	72	24	43
Average degree	2.118	2.938	2.471	3.625	0.706	1.344
Network diameter	4	4	4	3	3	3
Network density	0.064	0.095	0.075	0.117	0.021	0.043
Average clustering coefficient	0.353	0.487	0.591	0.648	0.05	0.119

### Contact

**Fig 2:**
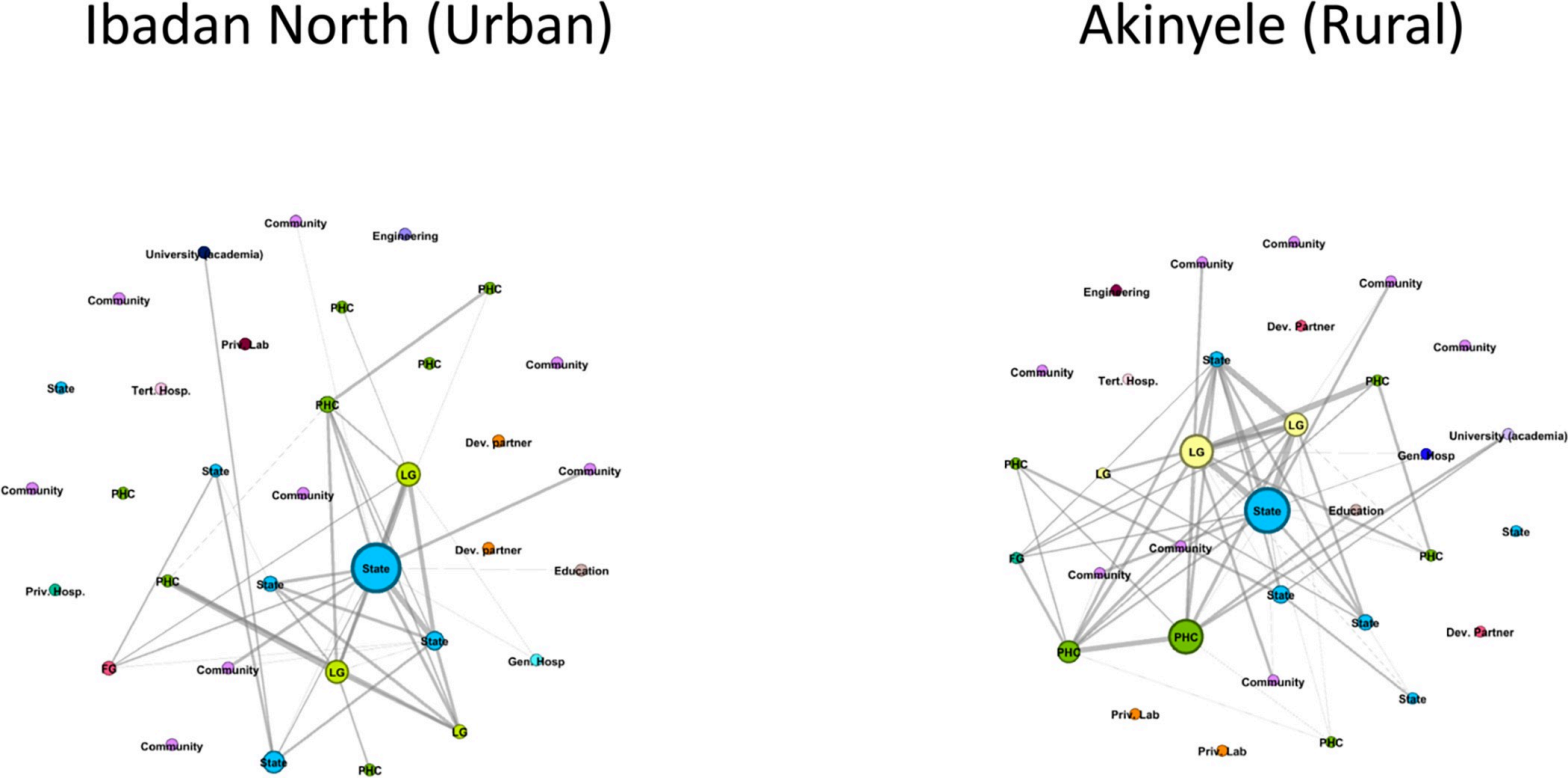
Betweenness centrality output (collaboration network). Key: LG: local government, FG: Federal Government, State: state government, PHC: Primary Health care centre, Priv. hosp: private hospital, Priv lab: private lab, Dev Partner: Developmental partner, Tert. Hosp: Tertiary hospital, Gen Hosp: General hospital (secondary care).

Figs 1 and 2 shows the contact patterns in the 2 LGAs from the lowest to the highest organizational level. Most of the contact activities were between stakeholders within the governance (local and state) and healthcare sector in both LGAs. There was no contact activity with community-level stakeholders and developmental partners.

### Linkage/collaboration

Within the linkage/collaboration networks, collaboration was strong between the state and local government governance structure. Other collaborating stakeholders were public healthcare stakeholders, especially the primary healthcare centres (PHC). There were no collaboration links between developmental partners and other stakeholders at the state and local government levels.

### Resource support

The resources support network showed similar patterns to the contact and collaboration networks. There was strong resource support between local and state governance structures, with some resource support between the Primary health care (PHC) centres and the federal government.

## Discussion

We applied social network analysis to the schistosomiasis control program stakeholder networks in Oyo state using 2 local governments as examples. The social network analysis measured the strength of contact/communication, collaboration and resource support networks over a 1-year period. Similar network structures were observed in both rural and urban LGAs. However, the rural local government appeared more active. The network structures displayed properties such as high clustering and low density. We will be analysing the three networks from three perspectives; whole network, stakeholder position and contextual.

From the whole network point of view, the three networks appear sparse (low density) with low levels of connections between all important stakeholders (Fig 1). Low network density can affect the effectiveness and efficiency of the stakeholders in meeting their goals, in this case, schistosomiasis elimination because of the sparsity of connections between stakeholders. Some studies have highlighted the importance of network density on network efficiency and effectiveness [24,26]. However, other studies argue that low density does not necessarily mean low efficiency since a network of strong and highly active stakeholders is not very effective [27–30]. This argument does not apply in this case as the network is skewed towards some categories of stakeholders who are driving the activities seen within the schistosomiasis control stakeholder network with an absence of weak or strong ties to other clusters of stakeholders. Weak ties are important because they serve as bridges between different clusters of stakeholders and are important for the rapid spread of information and innovation [30]. Due to the absence of weak and or strong ties, innovative practices, products and plans geared towards eliminating schistosomiasis may not spread through the networks leading to a lack of ownership by the community and other important stakeholders.

An in-depth look at the three networks shows differences between the contact, collaboration and resource support networks. The linkage/collaboration network highlights the strength of the relationship between the stakeholders and appears to be the strongest network (Fig 1 and Table 4). The contact network highlights the contact/communication activities between the stakeholders and appears to be the second most active network with the resource support network being the least active. The contact network confirms the that there is truly some collaboration between the stakeholders because collaboration activities do require contact between stakeholders. The resource support network is small indicating that resources (Table 2) are not readily available and or shared between the stakeholders. This implies that collaboration between the active stakeholders is mainly a formality due to their expected roles from set policies. For instance, it is expected that the LG NTD officer submits monthly reports to the state NTD officer as part of the responsibilities of the officer. It is known that active networks with shared resources are more collaborative and innovative [31]. Lack of important shared resources such as ideas, training and materials means that the network may not be open to new ideas and innovative practices which may impact reaching the schistosomiasis elimination goal of 2030.

From the stakeholder level perspective across all three networks (Figs 1 and 2), it is clear that stakeholder relationships are strongest between the organized governance and health sector with actors at the state, local government, federal government and primary care actors being the dominant stakeholders. This shows that the government at all levels (local government, state and federal) are the strongest players in the schistosomiasis control program and are the main drivers of change. In other to meet the WHO schistosomiasis elimination goals of 2030, other stakeholders such as community-level stakeholders and patient groups must be involved to increase community cooperation and accelerate disease elimination [1]. For schistosomiasis to be eliminated within this context, other stakeholders outside of the organized governance system must be active either within their clusters or in connection with other strong stakeholders within other clusters.

Another interesting finding from the stakeholder perspective is the gap between the development partners and organized governance system both at the state and local government levels (Figs 1 and 2). It is well known that developmental partners such as the WHO and other Non-Governmental Organizations provide technical support to countries both at the national and sub-national levels [1,32]. However, this support appears absent in this instance. Although support is mainly given to states which have a very high prevalence of schistosomiasis, Oyo state has a moderate incidence and it is expected that some forms of support in terms of resources, collaboration and contact activities should be seen in this context. Lack of collaboration between these important stakeholders means that meeting the schistosomiasis control goals, and the creation and diffusion of innovative practices and products may be limited or non-existent.

Another gap identified is the absence of relationships between stakeholders in public healthcare and the private healthcare sector. In addition, there is also no relationship between the organized health governance system (local, state and federal) and the private healthcare sector. There appears to be a parallelity in the organization of the healthcare system. This gap implies that stakeholders in the private healthcare system especially private laboratories do not necessarily have to report cases of schistosomiasis and are not involved in the data collection, reporting and training on schistosomiasis control. Consequently, cases of schistosomiasis may be largely underreported and this issue requires urgent policy action. It is known that the private sector is innovative [33], and involvement of this sector in schistosomiasis control may drive the development of innovative practices and may be one of the missing links in achieving schistosomiasis elimination.

The contextual perspective focuses on the network and actor positional differences between the urban and rural contexts in this research. The stakeholders within the rural LGA (Akinyele) are more active across the three networks compared with stakeholders in the Urban LGA(Ibadan North). There are several reasons for this pattern. First, schistosomiasis appears more dominant in rural communities where there is a lack of access to potable water and reliance on natural bodies of water which can be easily contaminated [3,34]. As such, these stakeholders are more likely to anticipate schistosomiasis infections thereby making more contact, collaborative actions and sharing more resources. Second, due to the sparse clustering of communities within rural areas, stakeholders within this context may rely more on communication, collaboration for information sharing and resource support such as training and ideas to reach their goals invariably leading to a more active network. Finally, due to the challenging topography of many rural areas, strong collaboration is critical in meeting policy-stipulated activities, and stakeholders within rural networks may rely strongly on community-level stakeholders for information. Evidence of this is seen in Figs 1 and 2.

It is important to emphasise that the peculiarities of the context such as cultural, political, physical and historical factors can affect stakeholder relationships and these should be strongly taken into account when working towards schistosomiasis control, and driving the adoption of innovative practices and products for schistosomiasis elimination.

### Limitations

This study is the only study to use SNA to measure the schistosomiasis control program network cohesion. Several limitations are noted that may impact the quality of the data. First, this study was cross-sectional and the data was collected in 2021 during the Covid-19 pandemic. It is possible that relationships between stakeholders dwindled during this period due to the focus of the health system on the pandemic. However, the effect of the pandemic on NTD activities was limited within the country and the streamlining of Covid-19 prevention activities into the NTD control activities ensured that NTD stakeholders were active during the research period [35]. Second, stakeholders interviewed gave a self-report of relationships which may be subject to over or under-reporting. Since trust has been built by prior relationships and interactions with the stakeholders [8,36], a true picture of current relationships was likely given. In addition, the use of a network roster which listed all stakeholders in combination with a snowball approach minimised under and over-reporting. Finally, this study was conducted in one state using the examples of 2 LGAs and cannot be generalised to other states and LGAs dues to differing stakeholder relationships. However, it can give insight into the picture of stakeholder relationships in similar settings in Nigeria, other developing country settings and across similar NTDs. This study can provide a baseline for other studies to measure collaboration within the schistosomiasis control program before implementing innovation or adopting the WHO NTD elimination plan.

## Conclusion

This study has highlighted gaps in the stakeholder relationships within the schistosomiasis control program in a state in Southwestern Nigeria and its implication for meeting the WHO schistosomiasis elimination goals of 2030. The limited cohesion in stakeholder relationships across the contact, collaboration and resource-sharing networks can limit the progress recorded in schistosomiasis control and may be the missing link in reaching the schistosomiasis elimination goals promptly. Improved contact, collaboration and resource sharing across all layers of stakeholders can provide benefits such as improved capacity, responsiveness, innovation and openness to new ways of achieving set goals. This study also provides baseline data for interventions targeting improved collaboration among stakeholders in the schistosomiasis control network in Nigeria.

Further research is needed to map and understand stakeholder relations within the NTD context in Nigeria and Africa. Identifying weak links within NTD relational network can give insights into challenges and gaps with disease elimination and offers an opportunity to strengthen relational ties and cohesion among NTD stakeholders.

## Supporting information

S1 File Network datasets for 2 local government areas. (XLSX)

## References

[1] World Health Organization. Ending the neglect to attain the Sustainable Development Goals–A road map for neglected tropical diseases 2021–2030. [Internet]. World Health Organization; 2020 [cited 2022 Oct 6]. Available from: https://www.who.int/publications/i/item/9789240010352

[2] WHO. Prevention and control of schistosomiasis and soil-transmitted helminthiasis: Technical Report Series N°912 [Internet]. World Health Organization; 2002 [cited 2022 Sep 19]. Available from: https://www.who.int/publications-detail-redirect/WHO-TRS-912 12592987

[3] Aula OP, McManus DP, Jones MK, Gordon CA. Schistosomiasis with a Focus on Africa. *Trop Med Infect Dis*. 2021 Jun 22;6(3):109. doi: 10.3390/tropicalmed6030109 34206495 PMC8293433

[4] Ogongo P, Nyakundi RK, Chege GK, Ochola L. The Road to Elimination: Current State of Schistosomiasis Research and Progress Towards the End Game. *Front Immunol*. 2022;13:846108. doi: 10.3389/fimmu.2022.846108 35592327 PMC9112563

[5] Petkovic J, Riddle A, Akl EA, Khabsa J, Lytvyn L, Atwere P, et al. Protocol for the development of guidance for stakeholder engagement in health and healthcare guideline development and implementation. *Syst Rev*. 2020 Feb 1;9(1):21. doi: 10.1186/s13643-020-1272-5 32007104 PMC6995157

[6] Masefield SC, Msosa A, Chinguwo FK, Grugel J. Stakeholder engagement in the health policy process in a low income country: a qualitative study of stakeholder perceptions of the challenges to effective inclusion in Malawi. *BMC Health Serv Res*. 2021 Sep 18;21(1):984. doi: 10.1186/s12913-021-07016-9 34537033 PMC8449519

[7] Neil J. Stakeholder Engagement: A Road Map to Meaningful Engagement. Doughty Centre, Cranfield School of Management; 2009 Aug. (How to do Corporate Responsibility). Report No.: 2.

[8] Onasanya A, Keshinro M, Oladepo O, Van Engelen J, Diehl JC. A Stakeholder Analysis of Schistosomiasis Diagnostic Landscape in South-West Nigeria: Insights for Diagnostics Co-creation. *Front Public Health.* 2020;8:697. doi: 10.3389/fpubh.2020.564381 33194966 PMC7661745

[9] Banda GT, Deribe K, Davey G. How can we better integrate the prevention, treatment, control and elimination of neglected tropical diseases with other health interventions? A systematic review. *BMJ Glob Health*. 2021 Oct 1;6(10):e006968. doi: 10.1136/bmjgh-2021-006968 34663634 PMC8524265

[10] Celik S. On the Paradoxical Nature of Innovation Evidence from Social Networks in Fryslân. [Netherlands]: Delft University of Technology; 2018.

[11] Zedan S, Miller W. Using social network analysis to identify stakeholders’ influence on energy efficiency of housing. *Int J Eng Bus Manag*. 2017 Jan 1;9:1847979017712629.

[12] Barabasi AL. Linked: How everything is connected to everything else and what it means for business, science, and everyday life. New York, NY: Penguin Group; 2003.

[13] Hanneman R, Riddle M. Introduction to social network methods. [Internet]. Riverside, CA: University of California, Riverside; 2005. Available from: https://faculty.ucr.edu/~hanneman/nettext/index.html

[14] Magnani M, Micenková B, Rossi L. Combinatorial Analysis of Multiple Networks. ArXiv [Internet]. 2013 Mar 20 [cited 2023 Feb 22]; Available from: https://www.semanticscholar.org/paper/Combinatorial-Analysis-of-Multiple-Networks-Magnani-Micenkov%C3%A1/038f34c6af7c0c1fcea96d3061ea13bd7440d8d2

[15] Cunningham FC, Ranmuthugala G, Plumb J, Georgiou A, Westbrook JI, Braithwaite J. Health professional networks as a vector for improving healthcare quality and safety: a systematic review. *BMJ Qual Saf*. 2012 Mar;21(3):239–49. doi: 10.1136/bmjqs-2011-000187 22129933 PMC3285140

[16] De Brún A, McAuliffe E. Social Network Analysis as a Methodological Approach to Explore Health Systems: A Case Study Exploring Support among Senior Managers/Executives in a Hospital Network. *Int J Environ Res Public Health.* 2018 Mar;15(3):511. doi: 10.3390/ijerph15030511 29534038 PMC5877056

[17] Smit LC, Dikken J, Schuurmans MJ, de Wit NJ, Bleijenberg N. Value of social network analysis for developing and evaluating complex healthcare interventions: a scoping review. *BMJ Open*. 2020 Nov 1;10(11):e039681. doi: 10.1136/bmjopen-2020-039681 33203632 PMC7674094

[18] Valente TW, Palinkas LA, Czaja S, Chu KH, Brown CH. Social Network Analysis for Program Implementation. *PLOS ONE.* 2015 Jun 25;10(6):e0131712. doi: 10.1371/journal.pone.0131712 26110842 PMC4482437

[19] Chambers D, Wilson P, Thompson C, Harden M. Social network analysis in healthcare settings: a systematic scoping review. *PloS One.* 2012;7(8):e41911. doi: 10.1371/journal.pone.0041911 22870261 PMC3411695

[20] Hu H, Yang Y, Zhang C, Huang C, Guan X, Shi L. Review of social networks of professionals in healthcare settings—where are we and what else is needed? *Glob Health.* 2021 Dec 4;17(1):139. doi: 10.1186/s12992-021-00772-7 34863221 PMC8642762

[21] Purington A, Stupp E, Welker D, Powers J, Banikya-Leaseburg M. Using Social Network Analysis to Strengthen Organizational Relationships to Better Serve Expectant and Parenting Young People. *Matern Child Health J.* 2020 Sep 1;24(2):232–42. doi: 10.1007/s10995-020-02992-6 32889682 PMC7497387

[22] Janse van Rensburg A, Petersen I, Wouters E, Engelbrecht M, Kigozi G, Fourie P, et al. State and non-state mental health service collaboration in a South African district: a mixed methods study. *Health Policy Plan.* 2018 May 1;33(4):516–27. doi: 10.1093/heapol/czy017 29462292

[23] Mukinda FK, Van Belle S, Schneider H. Local Dynamics of Collaboration for Maternal, Newborn and Child Health: A Social Network Analysis of Healthcare Providers and Their Managers in Gert Sibande District, South Africa. *Int J Health Policy Manag.* 2021 Sep 8;11(10):2135–45. doi: 10.34172/ijhpm.2021.106 34523867 PMC9808286

[24] Muthathi IS, Kawonga M, Rispel LC. Using social network analysis to examine inter-governmental relations in the implementation of the Ideal Clinic Realisation and Maintenance programme in two South African provinces. *PLOS ONE.* 2021 May 12;16(5):e0251472. doi: 10.1371/journal.pone.0251472 33979415 PMC8115818

[25] Soi C, Shearer J, Chilundo B, Muchanga V, Matsinhe L, Gimbel S, et al. Global health systems partnerships: a mixed methods analysis of Mozambique’s HPV vaccine delivery network actors. *BMC Public Health*. 2020 Jun 5;20(1):862. doi: 10.1186/s12889-020-08958-1 32503479 PMC7275554

[26] Provan KG, Milward HB. A Preliminary Theory of Interorganizational Network Effectiveness: A Comparative Study of Four Community Mental Health Systems. *Adm Sci Q.* 1995;40(1):1–33.

[27] Carroll N, Richardson I. Mapping a Careflow Network to assess the connectedness of Connected Health. *Health Informatics J.* 2019 Mar 1;25(1):106–25. doi: 10.1177/1460458217702943 28438102

[28] Hua L, Yang Z, Shao J. Impact of network density on the efficiency of innovation networks: An agent-based simulation study. *PLoS ONE.* 2022 Jun 17;17(6):e0270087. doi: 10.1371/journal.pone.0270087 35714137 PMC9205519

[29] Hua L, Wang W. The impact of network structure on innovation efficiency: An agent-based study in the context of innovation networks—ProQuest. Complexity [Internet]. 2015; 21: 111–122. [cited 2022 Oct 5];21(2). Available from: https://www.proquest.com/openview/286c14f24984027bd247e54baa19f48e/1?pq-origsite=gscholar&cbl=2029978

[30] Valente T. Social Networks and Health: Models, Methods, and Applications [Internet]. New York, NY: Oxford Academic; 2010 [cited 2022 Aug 13]. Available from: 10.1093/acprof:oso/9780195301014.001.0001

[31] van den Hoed MW, Backhaus R, de Vries E, Hamers JPH, Daniëls R. Factors contributing to innovation readiness in health care organizations: a scoping review. *BMC Health Serv Res.* 2022 Aug 5;22:997. doi: 10.1186/s12913-022-08185-x 35932012 PMC9354428

[32] World Health Organization. *A Roadmap for Implementation: accelerating work to overcome the global impact of neglected tropical diseases*. Geneva: World Health Organization; 2012.

[33] Bhattacharyya O, Khor S, McGahan A, Dunne D, Daar AS, Singer PA. Innovative health service delivery models in low and middle income countries—what can we learn from the private sector? *Health Res Policy Syst.* 2010 Jul 15;8(1):24. 20630108 10.1186/1478-4505-8-24 PMC3236300

[34] Adenowo AF, Oyinloye BE, Ogunyinka BI, Kappo AP. Impact of human schistosomiasis in sub-Saharan Africa. *Braz J Infect Dis*. 2015 Mar 1;19(2):196–205. doi: 10.1016/j.bjid.2014.11.004 25636189 PMC9425372

[35] McKay S, Shu’aibu J, Cissé A, Knight A, Abdullahi F, Ibrahim A, et al. Safely resuming neglected tropical disease control activities during COVID-19: Perspectives from Nigeria and Guinea. *PLoS Negl Trop Dis.* 2021 Dec 20;15(12):e0009904. doi: 10.1371/journal.pntd.0009904 34928945 PMC8687572

[36] Van GY, Onasanya A, van Engelen J, Oladepo O, Diehl JC. Improving Access to Diagnostics for Schistosomiasis Case Management in Oyo State, Nigeria: Barriers and Opportunities. *Diagnostics*. 2020 May;10(5):328. doi: 10.3390/diagnostics10050328 32443849 PMC7278006

